# ASO Author Reflections: Changes in the Urinary Microbiome After Transurethral Resection of Nonmuscle-Invasive Bladder Cancer: Insights from a Prospective Observational Study

**DOI:** 10.1245/s10434-024-15248-2

**Published:** 2024-04-11

**Authors:** Aleksander Ślusarczyk

**Affiliations:** Department of General, Oncological and Functional Urology, Medical University of Warsaw, Warsaw, Poland

## Past

The role of the urinary microbiome in developing and course of bladder cancer remains a matter of debate. Previous investigations had primarily focused on comparing urine microbiome composition between patients with and without evidence of tumor, overlooking the specific alterations induced by the transurethral resection of the bladder tumor (TURBT).^1^ Additionally, the unexplored territory of the tumor-associated microbiome remains understudied and its comparison to the microbiome detected in urine requires further studies, also regarding the urine method collection.^2,3^ Our study addressed the changes in the urinary microbiome after transurethral resection of non-muscle-invasive bladder cancer (NMIBC) and the characteristics of tumor tissue microbiome.^4^

## Present

Our findings revealed dynamic shifts in the bladder microbiome of low-grade Ta papillary NMIBC patients following TURBT. Notably, the phylogenetic diversity of the urine microbiome decreased at the 1-year follow-up, with distinct taxa enrichment compared with the time of index resection. Actinomyces, Candidatus, Sphingobacterium, Sellimonas, Fusobacterium, and Roseobacter were identified as the most differentially enriched taxa in the urine at follow-up. Furthermore, the tumor tissue microbiome displayed greater phylogenetic diversity than paired urine samples with enrichment of Acinetobacter and Sphingomonas taxa. Moreover, specific bacterial taxa are enriched in patients who will develop recurrence, namely Corynebacterium and Anaerococcus could serve as potential prognostic factors for future recurrence.

## Future

The implications of our study underscore the need for further research to unravel the interplay between bladder microbiome, tumor microbiome, and their alterations. Elucidating the predictive value of these microbiome changes could pave the way for personalized therapeutic interventions. Future studies should delve into the development of probiotic drugs, offering a targeted approach to modulate microbial communities and enhance antitumor immunity. A comprehensive understanding of the urinary microbiome's intricate relationship with disease recurrence and progression holds promise for more effective and personalized therapeutic strategies in the management of NMIBC.
